# Effects of Training on Lateralization for Simulations of Cochlear Implants and Single-Sided Deafness

**DOI:** 10.3389/fnhum.2018.00287

**Published:** 2018-07-17

**Authors:** Fei Yu, Hai Li, Xiaoqing Zhou, XiaoLin Tang, John J. Galvin III, Qian-Jie Fu, Wei Yuan

**Affiliations:** ^1^Department of Otolaryngology, Southwest Hospital, Third Military Medical University, Chongqing, China; ^2^House Ear Institute, Los Angeles, CA, United States; ^3^Department of Head and Neck Surgery, David Geffen School of Medicine, University of California, Los Angeles, Los Angeles, CA, United States

**Keywords:** cochlear implants, single-sided deafness, localization, lateralization, insertion depth

## Abstract

While cochlear implantation has benefitted many patients with single-sided deafness (SSD), there is great variability in cochlear implant (CI) outcomes and binaural performance remains poorer than that of normal-hearing (NH) listeners. Differences in sound quality across ears—temporal fine structure (TFS) information with acoustic hearing vs. coarse spectro-temporal envelope information with electric hearing—may limit integration of acoustic and electric patterns. Binaural performance may also be limited by inter-aural mismatch between the acoustic input frequency and the place of stimulation in the cochlea. SSD CI patients must learn to accommodate these differences between acoustic and electric stimulation to maximize binaural performance. It is possible that training may increase and/or accelerate accommodation and further improve binaural performance. In this study, we evaluated lateralization training in NH subjects listening to broad simulations of SSD CI signal processing. A 16-channel vocoder was used to simulate the coarse spectro-temporal cues available with electric hearing; the degree of inter-aural mismatch was varied by adjusting the simulated insertion depth (SID) to be 25 mm (SID25), 22 mm (SID22) and 19 mm (SID19) from the base of the cochlea. Lateralization was measured using headphones and head-related transfer functions (HRTFs). Baseline lateralization was measured for unprocessed speech (UN) delivered to the left ear to simulate SSD and for binaural performance with the acoustic ear combined with the 16-channel vocoders (UN+SID25, UN+SID22 and UN+SID19). After completing baseline measurements, subjects completed six lateralization training exercises with the UN+SID22 condition, after which performance was re-measured for all baseline conditions. Post-training performance was significantly better than baseline for all conditions (*p* < 0.05 in all cases), with no significant difference in training benefits among conditions. Given that there was no significant difference between the SSD and the SSD CI conditions before or after training, the results suggest that NH listeners were unable to integrate TFS and coarse spectro-temporal cues across ears for lateralization, and that inter-aural mismatch played a secondary role at best. While lateralization training may benefit SSD CI patients, the training may largely improve spectral analysis with the acoustic ear alone, rather than improve integration of acoustic and electric hearing.

## Introduction

While cochlear implantation has been shown to improve sound source localization in patients with single-sided deafness (SSD), there is great variability in localization performance among SSD cochlear implant (CI) patients (Vermeire and Van de Heyning, 2009; Arndt et al., 2011; Firszt et al., 2012; Kamal et al., 2012; Távora-Vieira et al., 2013; Tokita et al., 2014; van Zon et al., 2015; Dorman et al., 2016). Localization performance for SSD CI patients remains poorer than that of normal-hearing (NH) listeners and is comparable to that of bilateral CI patients (Dorman et al., 2016). The poorer performance and variability may reflect difficulties in combining temporal fine structure (TFS) cues from acoustic hearing with relatively coarse spectro-temporal envelope cues from electric hearing. Clinical fitting of the CIs for SSD patients is similar to that of unilateral CI patients, in that a wide acoustic frequency range is mapped onto the (typically) limited cochlear extent of the electrode array. Depending on the length of the array, the insertion depth, and the pattern of nerve survival (all of which comprise the electrode-neural interface), CI patients often experience some degree of intra-aural frequency mismatch between the acoustic input and the electrode place of stimulation. SSD CI users also often experience some degree of inter-aural mismatch between frequency information delivered to the acoustic and CI ears. While CI users are able to partly adapt to intra- and inter-aural frequency mismatch as they gain experience with their device (Fu et al., 2002; Svirsky et al., 2004, 2015; Reiss et al., 2007, 2008, 2012, 2015; Vermeire et al., 2015), adaptation may not be complete. It is possible that explicit training may help SSD CI patients better integrate acoustic and electric hearing and improve localization performance.

For NH listeners, inter-aural level differences (ILDs) and inter-aural timing differences (ITDs) can be used to localize sounds. Due to interactions between frequency wavelength and head size, ILDs are high-frequency cues (>0.6 kHz). Due to limits of temporal processing, ITDs are low-frequency cues (<0.5 kHz). For low-frequency sounds, ILDs are often unavailable (Shinn-Cunningham, 2000), and NH listeners localize using ITDs. Because TFS cues are unavailable with electric hearing, and because stimulation patterns are not synchronized across ears, bilateral and SSD CI patients primarily use ILDs to lateralize sounds (Aronoff et al., 2010; Dorman et al., 2015). ITDs require precise processing of temporal cues at characteristic frequencies for neurons for the lower and medial superior olive (Grothe et al., 2010). Inter-aural mismatch has been shown to limit access to ITDs in bilateral and SSD CI patients. Psychophysical studies have shown that bilateral CI users’ perception of ITDs is sharply reduced for small inter-aural mismatches (Long et al., 2003; Poon et al., 2009). Goupell et al. (2013) evaluated the effects of inter-aural frequency mismatch on binaural processing in NH listeners using band-limited pulse trains. They found that just-noticeable differences (JNDs) for ITD and ILDs increased with decreasing bandwidth and increasing mismatch. Kan et al. (2015) also found that ITD and ILD JNDs worsened with increasing amounts of inter-aural mismatch.

While inter-aural frequency mismatch may be reduced by modifying the acoustic-to-electric frequency allocation, CI patients are able to at least partially adapt to mismatch (Reiss et al., 2015; Svirsky et al., 2015). Svirsky et al. (2015) evaluated whether adults can adapt to sharply different frequency-to-place maps across ears. They measured inter-aural electrode pitch ranking and listener-driven selection of the frequency allocation that produced the highest intelligibility in two bilateral CI patients who had a full electrode insertion in one ear and a much shallower insertion in the other ear but were given the same clinical frequency allocations in both ears. They found that both listeners showed substantial but incomplete adaptation for the ear with the shallower insertion, even after extended experience. Similarly, Reiss et al. (2011) measured pitch perception in a CI subject with a 10-mm electrode array in one ear and a 24-mm electrode array in the other ear. Both processors were programmed with the same input frequency range of 188–7938 Hz, despite the large differences in electrode length and insertion depth. After 2–3 years of experience, pitch-matched electrode pairs between CIs were aligned closer to the processor-provided frequencies than to cochlear position. They suggested that pitch perception may have adapted to reduce the perceived inter-aural mismatch, despite the 2–3 octave difference in terms of cochlear place of stimulation between ears. Eapen et al. (2009) reported that bilateral CI patients’ localization and spatial segregation of speech and noise continued to improve long after initial activation of both implants. Binaural performance for SSD CI patients has been shown to continue to improve more than 3 years after implantation (Gartrell et al., 2014; Mertens et al., 2017).

While long-term experience may improve bilateral and SSD CI patients’ localization, explicit training may further improve and/or accelerate adaptation to inter-aural mismatch. Many CI studies have shown that auditory training can improve speech and music perception, even after years of experience with a device and/or signal processing strategy (Fu et al., 2004, 2015; Fu and Galvin, 2007, 2008; Galvin et al., 2007, 2012; Stacey et al., 2010; Oba et al., 2011) and in NH subjects listening to CI simulations (Rosen et al., 1999; Fu et al., 2005b; Faulkner, 2006; Stacey and Summerfield, 2007, 2008). However, there are very few training studies related to spatial hearing, such as sound localization, in CI patients with bilateral inputs. Tyler et al. (2010) reported spatial training data in three bilateral CI subjects who were trained in the lab or at home using an 8-speaker array. Results showed better localization and speech perception in noise after training. Note that this approach to training (training in sound field using a multi-speaker array in the home or laboratory) is not convenient. A different training approach would be to use direct audio input (DAI) to the CI speech processor and/or insert earphones to the acoustic hearing ear with head-related transfer functions (HRTFs), as used for testing lateralization in bilateral CI patients in previous studies (Chan et al., 2008; Aronoff et al., 2010).

SSD CI patients must adapt to the differences between acoustic and electric stimulation as well as to inter-aural mismatch. In this study, lateralization training was evaluated in NH subjects listening to simulations of SSD CI processing (acoustic hearing in one ear, 16-channel vocoder in the other ear); lateralization was also measured with the acoustic hearing in one ear only to simulate SSD performance before implantation. Note that in this study, “CI simulation” is a term of convenience, as is not intended to convey the veracity of simulated electric hearing. While many previous studies have used “CI simulations”, vocoder processing is used to create conditions of spectral degradation, channel interaction, and frequency mismatch that are thought to limit real CI performance. As such, CI simulations may sometimes produce similar performance to that of real CI patients, but do not capture the quality of electric hearing (Dorman et al., 2017). For the 16-channel vocoder, different electrode insertion depths were simulated to introduce different degrees of inter-aural mismatch. After completing baseline measurements, subjects were training with one of the SSD CI simulations, after which performance for all conditions was remeasured. We hypothesized three possible training outcomes: (1) better post-training lateralization for all conditions including the monaural SSD condition, indicating improved perception of spectral/head shadow cues in the NH ear, rather than improved integration of acoustic and electric hearing; (2) better post-training performance for the SSD CI simulations but not for the SSD simulation, indicating better integration of acoustic and electric cues; or (3) better post-training performance for the trained SSD CI simulation but not for the untrained SSD CI simulations, indicating adaptation to a specific inter-aural mismatch.

## Materials and Methods

### Subjects

Twelve (eight males and four females; mean age = 27 years, age range = 25–30 years) young adult NH native speakers of Mandarin Chinese participated in the study. All subjects had pure-tone thresholds ≤20 dB HL for audiometric frequencies 250, 500, 1000, 2000, 4000 and 8000 Hz. All reported speaking, reading, and writing Chinese with excellent proficiency in terms of daily communication. Exclusion criteria included organic brain diseases and other physical or mental illness that could lead to cognitive impairment. In compliance with ethical standards for human subjects, written informed consent was obtained from all participants in accordance with the Declaration of Helsinki before proceeding with any of the study procedures. This study was approved by Institutional Review Board in Department of Otolaryngology, Southwest Hospital, Third Military Medical University, Chongqing, China.

### Sound Lateralization

Lateralization was measured using headphones that incorporated a HRTF, as in previous studies (Chan et al., 2008; Aronoff et al., 2010). Subjects were asked to lateralize a broadband impulsive sound originating from one of twelve virtual locations. The sound sources were located behind the listener, spaced 15° apart. As described in Chan et al. (2008), the virtual sound sources were located behind the listener to reduce the sensation that sounds were not externalized, which can occur with HRTFs for the front half-field. The stimulus was a broadband impulse sound (gunshot) presented at 65 dBA; the presentation level from trial to trial was roved by 6 dB to reduce the availability of loudness cues for lateralization. Prior to formal testing in each condition, subjects were given a preview in which the stimulus was played from each of 12 sound source locations in order. During testing, a virtual sound source was randomly selected (without replacement) and the gunshot was presented from that source. The subject responded by clicking on one of loudspeakers shown on a computer screen that mirrored the virtual locations, after which a new stimulus was presented. Stimuli were presented twice from each sound source (24 trials in each test block). Lateralization accuracy was quantified in terms of the root mean square error (RMSE) across all virtual locations, as in previous SSD CI localization studies (Arndt et al., 2011; Firszt et al., 2012; Dorman et al., 2016). The square root of the squared difference (in degrees) between each target and response location was averaged across all target locations to calculate RMSE.

### Signal Processing

**Figure 1:**
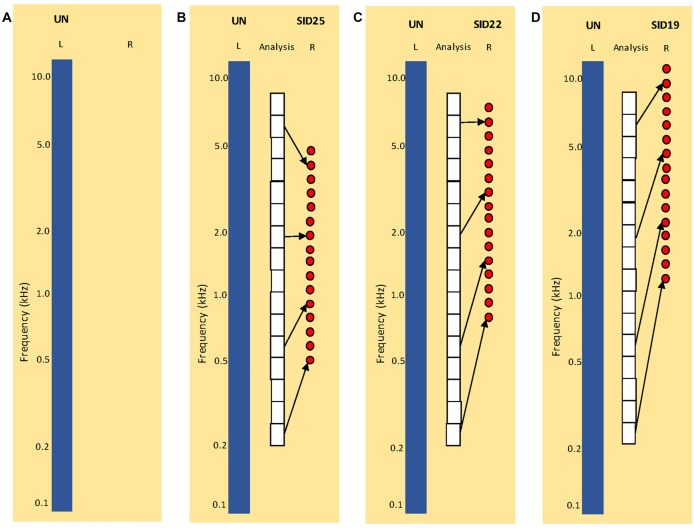
Illustration of experimental listening conditions. UN = unprocessed speech; SID = simulated insertion depth (in mm from the base) for the 16-channel vocoders). **(A)** Simulation of single-sided deafness (SSD; monaural listening with the left ear only). **(B)** Simulation of SSD cochlear implant (CI) with SID25. The white boxes show the frequency analysis bands. The left blue bar shows UN = unprocessed speech delivered to the left ear. The red circles show the center frequencies of the sine-wave carriers for the 16-channel vocoder delivered to the right ear. **(C)** Similar to **(B)**, but for SID22. **(D)** Similar to **(B,C)**, but for SID19. Note that the frequency analysis bands are the same for **(B–D)**.

Stimuli were processed by the HRTF before any subsequent signal processing. The MIT HRTF measured with a KEMAR dummy head^1^ was used for all NH listeners. To broadly simulate combined acoustic and electric hearing as might be experienced by SSD CI patients, original signals were delivered to left ear, and 16-channel vocoded signals were delivered to the right ear. The vocoder greatly reduced the spectral resolution, as might be experienced by CI listeners. The 16-channel sine-wave vocoder was implemented as in Fu et al. (2004, 2005b). First, the signal was processed through a high-pass pre-emphasis filter with a cutoff of 1200 Hz and a slope of −6 dB/octave. The input frequency range (200–7000 Hz) was then divided into 16 frequency bands, using 4th order Butterworth filters distributed according to Greenwood (1990) frequency-place formula. The temporal envelope from each band was extracted using half-wave rectification and low-pass filtering with a cutoff frequency of 160 Hz. The extracted envelopes were then used to modulate the amplitude of sinewave carriers. For the sine-wave carriers, the frequency extent was 16 mm, similar to electrode array lengths used in some commercial CI devices. Three simulated insertion depths (SIDs) were tested: 25 mm (SID25), 22 mm (SID22) and 19 mm (SID19), relative to the base. The carrier frequency ranges were 0.5–5.1 kHz for SID25, 0.9–7.8 kHz for SID22, and 1.4–11.8 kHz for SID19. The SIDs were selected to introduce different degrees of inter-aural mismatch for different frequency regions. For each SID condition, the sinewave carriers were distributed according to Greenwood (1990). Figure 1 illustrates the four experimental listening conditions: (A) UN (SSD; unprocessed signal to the left ear only), (B) UN+SID25 (unprocessed signal to the left ear, SID25 simulation to the right ear), (C) UN+SID22 (unprocessed signal to the left ear, SID22 simulation to the right ear) and (D) UN+SID19 (unprocessed signal to the left ear, SID19 simulation to the right ear). For UN+SID25, there was moderate frequency mismatch and compression at the basal and apical regions of the carrier range, with less mismatch in the middle region. For UN+SID22, there was substantial frequency mismatch and compression at the middle and apical regions of the carrier range, with less mismatch at the basal region. For UN+SID19, there was severe frequency mismatch and compression at the apical end of the carrier range, with substantial mismatch at the middle and basal regions. As a control condition, original speech was delivered to both the left and right ears (UN+UN).

### Test and Training Procedures

To familiarize subjects with the test procedures and to minimize procedural learning, lateralization was first measured for the UN+UN control condition (binaural lateralization with normal hearing in each ear); six familiarization test runs were completed. Next, baseline lateralization was measured for the SSD, UN+SID25, UN+SID22 and UN+SID19 conditions. Each of these conditions were tested one time, and the test order was randomized across subjects. Once baseline measurements were completed, subjects were trained while listening to the UN+SID22 listening condition. Training was similar to testing except that visual feedback was provided as to the correctness of response, and auditory feedback was provided in which the correct and incorrect sound sources were replayed. Six training runs were conducted in each subject before re-measuring lateralization for all listening conditions.

## Results

For the familiarization with the UN+UN control condition, some procedural learning was observed. Mean RMSE was reduced from 28.8° for Run 1 to 18.5° for Run 6. The individual data points for all subjects could be found in Supplementary Table S1. Because the distribution of the data was not normal, a one-way repeated measures analysis of variance (RM ANOVA) on ranked data was performed (Friedman test), with test run as the factor. Results showed a significant effect of test run (*χ*^2^ = 32.5; dF = 5; *p* < 0.001). *Post hoc* Tukey pairwise comparisons showed that RMSE for Runs 5 and 6 were significantly lower than for Runs 1 and 2 (*p* < 0.05 in all cases). No significant differences were observed among the remaining test runs. Asymptotic performance (across Runs 5 and 6) was 18.4°.

**Figure 2:**
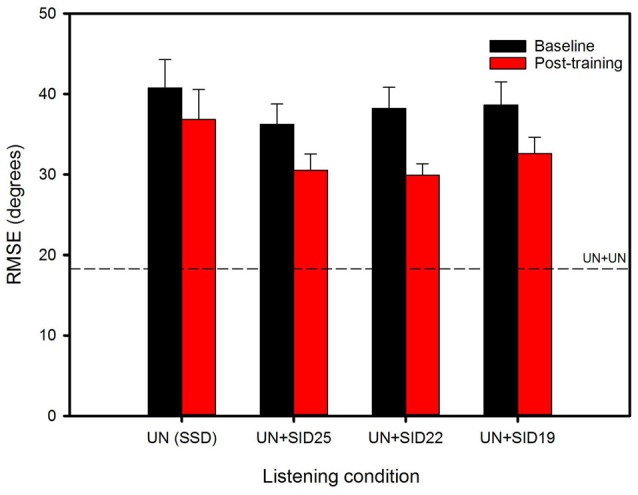
Mean RMSE for the experimental listening conditions, before (baseline) and after training with UN+SID22. The error bars show the standard error. The dashed line shows mean RMSE for the UN+UN control condition.

**Figure 3:**
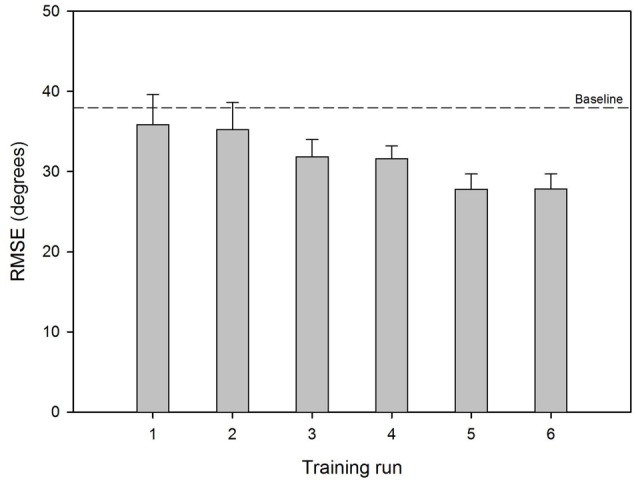
Mean RMSE across training runs with UN+SID22. The error bars show the standard error. The dashed line shows mean baseline RMSE for UN+SID22.

Figure 2 shows mean RMSE before and after training for the experimental listening conditions; performance for the UN+UN control condition is shown by the dashed line. Mean baseline RMSE was 40.8°, 36.2°, 38.2° and 38.7° for the SSD, UN+SID25, UN+SID22 and UN+SID19 conditions, respectively. After training with UN+SID22, the mean RMSE was reduced by 3.9°, 5.7°, 8.3° and 6.0° for the SSD, UN+SID25, UN+SID22 and UN+SID19 conditions, respectively. A two-way RM ANOVA was performed on the lateralization data, with listening condition (SSD, UN+SID25, UN+SID22, UN+SID19) and training (baseline, post-train) as factors. Results showed a significant effect for training [*F*_(1,33)_ = 24.1, *p* < 0.001], but not for listening condition [*F*_(3,33)_ = 1.2, *p* = 0.332]; there was no significant interaction [*F*_(3,33)_ = 1.2, *p* = 0.331]. A one-way RM ANOVA was also performed on the UN+UN data, with training (baseline, post-train) as the factor. Results showed no significant effect for training [*F*_(1,11)_ = 0.30, *p* = 0.597], suggesting that no procedural learning of the lateralization task had occurred as a result of training with UN+SID22. Figure 3 shows mean RMSE as a function of training run; baseline performance for UN+SID22 is shown by the dashed line. Mean RMSE was 38.2°, 35.9°, 35.3°, 31.9°, 31.6°, 27.8°, and 27.9° for baseline and training Runs 1–6, respectively. By Run 6, the mean RMSE was reduced by 10.3°. Because the distribution of training run data was not normal, a one-way RM ANOVA on ranked data was performed (Friedman test), with training run (baseline, Run 1, Run 2, Run 3, Run 4, Run 5, Run 6) as the factor. Results showed a significant effect of training run (*χ*^2^ = 49.8; dF = 6; *p* < 0.001). *Post hoc* Tukey pairwise comparisons showed that RMSE was significantly lower for training Runs 3–6 than for baseline and training Runs 1–2 (*p* < 0.05 in all cases), and significantly lower for training Runs 5–6 than for Runs 1–2 (*p* < 0.05 in all cases). There were no significant differences in RMSE among the remaining training runs.

## Discussion

The data show that binaural lateralization was much poorer with the SSD CI simulations than with original speech (UN+UN), consistent with localization data from real SSD CI patients (Dorman et al., 2016). Interestingly, there was no significant difference in lateralization between the monaural SSD and the binaural SSD CI simulations, suggesting that lateralization was driven by spectral analysis and head shadow for the acoustic hearing ear, with little integration of TFS and coarse-spectral envelope cues. Lateralization training improved performance for all conditions, with no significant difference among the monaural and binaural listening conditions, suggesting that the training mostly improved performance with the acoustic hearing ear.

### Lateralization vs. Localization

The present RMSE for lateralization was similar to that in Aronoff et al. (2010). However, significant procedural learning effects were observed for the UN+UN control condition, suggesting that the NH listeners needed four or more runs with original signals to adapt to the test procedure and/or the HRTF before achieving asymptotic performance. Note that asymptotic lateralization for the UN+UN condition (18.4°) was poorer than NH localization performance measured in sound field (e.g., 6° in Yost et al., 2013; Dorman et al., 2016; 12.4° in our unpublished data for the same stimuli using the physical 12-speaker setup). This suggests that lateralization with HRTFs may be poorer than localization in sound field. In Aronoff et al. (2010), there was no significant difference between lateralization measured with HRTFs and localization measured in sound field. Note that in this study and in Chan et al. (2008) and Aronoff et al. (2010), lateralization and/or localization were measured with the sound sources behind the listener. In Yost et al. (2013) and Dorman et al. (2016), localization was measured with the sound sources in front of the listener. As stated by Chan et al. (2008), true auditory-only localization requires that the sound sources not be visible to the subject. Placing the speakers behind the listener also minimized any tendencies to turn the head towards the sound source, which can greatly improve localization (Thurlow and Runge, 1967; Perrott et al., 1987; Feinkohl et al., 2013). Also, Chan et al. (2008) noted that for HRTFs, sound sources in the front field were not externalized as well as when sources were behind the listener. The HRTF and the virtual position of the speakers behind the listener may partly explain the discrepancy between binaural lateralization with original signals (UN+UN) in this study and localization in previous studies.

Interestingly, mean RMSE with one acoustic ear (SSD) was 40.8°, much better than the 68.0° observed for real SSD patients in Dorman et al. (2016). For both studies, the presentation level was roved by 4–6 dB around the target level of 65 dBA. It is possible that tinnitus or some other impairment may have affected monaural performance in SSD patients in Dorman et al. (2016), or that differences between localization and lateralization (with HRTFs) with different positioning of sound sources (in front or behind listeners) may explain differences in monaural performance. Note that lateralization performance was poorer for the present SSD CI simulations (mean RMSE = 37.7°) than localization performance for real SSD CI patients in (Dorman et al. (2016); mean RMSE = 28.0°). Again, differences between lateralization and localization may have contributed to the differences in RMSE. More likely, long-term experience with combined acoustic and electric hearing may have contributed to better localization. In this study, NH subjects had very limited experience combining TFS and coarse spectro-temporal envelope cues, as well as limited experience with monaural lateralization.

### Effects of Signal Degradation and Inter-aural Mismatch

Before or after training, binaural lateralization was much poorer when unprocessed speech (UN) was combined with 16-channel vocoders than when combined with UN cues in the opposite ear (UN+UN control condition). There was no significant difference in lateralization among the SSD and SSD CI simulations, suggesting that adding coarse spectro-temporal cues in the opposite ear did not benefit the NH subjects in this study. This finding is not in agreement with many studies with real SSD patients that show significant improvements in localization after cochlear implantation (Arndt et al., 2011; Hansen et al., 2013; Dorman et al., 2016; Mertens et al., 2016; Dillon et al., 2017). Note that in these SSD CI studies, there was considerable variability in localization performance after implantation. Also note that localization performance before implantation in SSD patients in these studies was generally much poorer than monaural lateralization in this study, allowing for greater room for improvement. The mean RMSE in this study across the SSD CI simulations (37.7°) was comparable to that of SSD patients in Dillon et al. (2017) at 1-month post-activation (37.0°). However, mean RMSE in Dillon et al. (2017) improved to 27.0° at 6 months post-activation, comparable to performance by experienced SSD CI patients in Dorman et al. (2016). Integration of acoustic and electric hearing may be different for real SSD CI patients and may depend on longer-term experience than the training provided in this simulation study.

The lack of significant difference among the different SSD CI simulations suggests that the degree of inter-aural mismatch was not a limiting factor in binaural localization. This finding is in contrast to previous studies with bilateral CI users that showed better ITD and ILD perception as the inter-aural frequency mismatch was reduced (Long et al., 2003; Poon et al., 2009; Goupell et al., 2013; Kan et al., 2015). Note that ITD and ILD thresholds were measured for single electrode pairs in those studies, as opposed to lateralization measured with a broadband stimulus in this study. It is also possible that sound quality differences between TFS cues in one ear and coarse spectro-temporal cues in the other may have been much greater than the degree of inter-aural mismatch among the SSD CI simulations. The present findings are also different from a related study by Zhou et al. (2017) using the same SSD CI simulations as in this study. In that study, speech understanding for spatially separated speech and noise worsened as the inter-aural frequency mismatch was increased. This is not surprising as a severe tonotopic mismatch in the CI simulation would negatively affect speech understanding with the CI ear. Lateralization does not require speech understanding, so the effects of inter-aural mismatch (or tonotopic mismatch in the CI ear) may not be as detrimental as for speech perception with spatial cues. Still, reducing interaural mismatch would be advantageous for spatial perception when both speech understanding and localization are considered.

The lack of significant difference between the SSD simulation and the SSD CI simulations suggests that monaural lateralization may have driven binaural performance. Some degree of monaural localization is possible using fine spectral analysis from one ear, although this is more useful in the vertical than in the horizontal plane (Blauert, 1997; Grothe et al., 2010). SSD patients may have long-term experience using such monaural spectral analysis to localize sounds. Liu et al. (2018) showed that monaural localization in SSD patients depended on the duration of deafness and the degree of tinnitus severity, with longer duration of deafness and no tinnitus associated with better localization. In this study, the mean RMSE was for monaural lateralization was 40.8° and 36.8° before and after the training, comparable to monaural localization by SSD subjects with more than 2 years of deafness (38.3°) or no tinnitus (38.8°) in Liu et al. (2018); mean RMSE was much higher for SSD patients with less than 1 year of deafness (55.9°) or tinnitus (50.5°) in Liu et al. (2018). As such, binaural performance was largely driven by monaural lateralization with UN and could be improved by long-term experience or training.

### Training Effects

As noted above, repeated testing (without feedback) of the UN+UN control condition revealed some procedural learning and/or adaptation to the HRTF. Baseline performance for the SSD and SSD CI simulations was only measured one time. It is possible that repeated testing might have also shown some procedural learning and/or adaptation to the simulations. Note that for all conditions, subjects were given a preview of all sound sources in sequence, which may have provided some familiarization with the signal processing.

The six training runs with UN+SID22 significantly improved lateralization for all experimental conditions. While the mean reduction in RMSE was larger for the trained UN+SID22 condition (8.3°) than for the untrained SSD (3.9°), UN+SID25 (5.7°) and UN+SID19 (6.0°) conditions, there was no significant difference in training benefits across conditions. However, training benefits were highly variable across subjects, with post-training reductions in RMSE ranging from −5.7° to 19.1°. The lack of significant difference in training benefit suggests that subjects may have largely attended to and improved perception of spectral cues with the NH ear alone. Training with the NH ear alone might have provided insight as to whether the present binaural training simply improved monaural lateralization; unfortunately, this was not done in this study. Thus, our first hypothesis regarding training—improved perception of spectral/head shadow cues in the NH ear alone—seems to be supported by the present data. It is possible that further training with UN+SID22 would have produced further improvements in lateralization. Note that RMSE was significantly lower than baseline by training Run 3, with no subsequent significant improvement relative to baseline. However, RMSE for training Runs 5–6 were significantly better than with Runs 1–2, suggesting some gradual improvement beyond training Run 3. Further testing with more extensive training might reveal significant adaptation to a trained inter-aural mismatch.

### Implications for SSD CI Patients

Different from the present results, SSD patients’ localization generally improves when the coarse spectro-temporal cues from the CI ear are combined with the TFS cues from the NH ear. However, in both the present and previous studies, SSD CI performance remains much poorer than binaural performance with two NH ears. The coarse spectro-temporal resolution of the CI and inter-aural mismatch may limit integration of acoustic and electric hearing, although the present data suggest that the coarse resolution may a major limiting factor, at least for multi-channel localization. SSD CI patients often have limited opportunity to adapt to electric hearing, given the dominance of the acoustic ear in everyday listening.

For SSD CI patients, adjustments to the clinical frequency allocation may help reduce inter-aural mismatch; depending on the degree of mismatch, optimization of the frequency allocation might result in information loss (e.g., low-frequency information might be discarded to tonotopically match the place of the most apical electrode). Thus, there is a tradeoff between reducing tonotopic mismatch and information loss that should be considered. The present data suggest that difficulties integrating acoustic TFS cues and coarse spectro-temporal envelope cues from the CI may be a major limiting factor. It is unclear whether training might help with this integration or improve analysis of spectral information from each ear to improve localization. The present results suggest that training may largely improve analysis with the acoustic ear, where TFS cues were available. It is possible that in the real CI case, where there is longer-term experience with electric hearing, training may also improve spectral analysis when only coarse spectral cues are available. Again, real SSD CI patients often show improved localization with combined acoustic and electric hearing, relative to acoustic hearing only. The NH subjects in this study may have had too little experience with the listening conditions for the training to show comparable benefits.

The present lateralization training could be easily implemented on home computers or mobile devices, allowing SSD CI patients (and bilateral CI patients) to improve spatial perception. Even if the training only improves monaural utilization of spectral cues in each ear (as suggested by the present data), SSD patients may be better able to use the coarse spectral cues from the CI ear for localization after training. In this study, a generic HRTF was used. Given differences in microphone placement across CI devices (and differences between microphone input and acoustic hearing with pinna), it is important that HRTFs be created for individual patients with regard to their CI device (Aronoff et al., 2011). It is also important that the HRTFs represent localization performance in sound field (as in Aronoff et al., 2010). If these issues can be resolved, computer-based lateralization training may benefit SSD CI patients’ localization performance.

Unfortunately, no objective measurements were collected in this study. In future studies, electroencephalography (EEG) data might show sensitivity to differences between acoustic and electric stimulation and to inter-aural mismatch. The binaural interaction component (BIC), defined as the difference (or ratio) between the binaural response and the sum of monaural responses from each ear, may provide insight into whether acoustic and electric stimulation (real or simulated) can be combined across ears, as well as effects of inter-aural mismatch on integration. Hu et al. (2016) found that the BIC could be used to match pairs of electrodes across ears in bilateral CI patients. Interestingly, there was no correlation between BIC matching and pitch-matching, suggesting that the objective measure may be more effective in identifying matched pairs of electrodes across ears. Zhang and Boettcher (2008) measured BIC for acoustic steady-state responses (ASSRs) to ILDs and ITDs in NH listeners. They found that the BIC was different for ILDs and ITDs, suggesting that these binaural cues may be processed by different auditory pathways. Zhang et al. (2018) also measured the BIC in SSD CI patients. They measured the acoustic change complex (ACC) for changes in pure-tone frequency. Presumably differences in spectral resolution would affect responses within each ear, and inter-aural mismatch would affect binaural responses. They found that responses from the NH ear had larger amplitude and shorter latencies than responses from the CI ear. Zhang et al. (2018) also found that the binaural interaction ratio (binaural response/sum of monaural responses) was similar between NH and SSD CI listeners, suggesting that binaural interaction may be similar across listener groups, despite absolute differences in response amplitude and latency. In this study, adding the 16-channel vocoder to the NH ear did not significantly change localization performance, regardless of the degree of inter-aural mismatch. As such, it is as yet unclear how objective measurements such as BIC may relate to localization performance in SSD CI listeners.

### Limitations of the Study

There are also some limitations to this study that should be considered. First, the 16-channel vocoders likely “simulate” only limited aspects of electric hearing, namely coarse spectro-temporal resolution and different degrees of acoustic-to-electric frequency mismatch. Other aspects of electric hearing (e.g., compression of the acoustic input onto the electric dynamic range, interaction between electrodes in terms of current spread/spread of excitation, non-uniform nerve survival, fixed-rate pulse-train stimulation, etc.) were not simulated, and they may contribute to acoustic-electric integration in real SSD CI patients. Second, the use of a single generic HRTF may not be appropriate for all the NH subjects in this study and may not extrapolate to SSD CI patients who must combine acoustic hearing with pinna affects with electric hearing via microphone input. Third, the very limited training performed in this study may not be comparable to long-term experience with acoustic-electric hearing in SSD CI patients. And finally, monaural hearing in NH subjects (the SSD simulation) may not be comparable to that in SSD patients, which may be affected by the duration of deafness and/or the severity of tinnitus. Still, the present data suggest that, if properly implemented, lateralization training on home computers or mobile devices may benefit SSD CI patients.

## Conclusion

In this study, lateralization was measured in NH subjects listening to SSD and SSD CI simulations before and after training. In the SSD CI simulations, 16-channel vocoders were used to degrade spectro-temporal cues and the degree of inter-aural mismatch was varied across different SIDs. Training with one of the SSD-CI simulations significantly improved performance for all the SSD CI simulations as well as for the SSD simulation (monaural listening with the NH ear alone). There was no significant difference before or after training among the SSD and SSD CI simulations, suggesting that performance was largely driven by spectral analysis with the NH ear alone. The degree of inter-aural mismatch did not significantly affect binaural lateralization, suggesting that difficulty integrating TFS cues with coarse spectro-temporal cues was a major limiting factor in this study. Different from real SSD CI patients who often exhibit a binaural benefit for localization after implantation, the present NH subjects may have had insufficient experience with the SSD CI simulations to experience a binaural benefit with training.

## Author Contributions

WY and Q-JF designed the study. WY, Q-JF, FY and HL conducted the experiment. FY, HL, XZ and XT performed experimental operation and data analysis. FY, HL and Q-JF prepared the manuscript. WY, JG and Q-JF supervised the whole research and revised the article. All authors approved the final version of the manuscript.

## Conflict of Interest Statement

The authors declare that the research was conducted in the absence of any commercial or financial relationships that could be construed as a potential conflict of interest.

## Footnotes

^1^http://sound.media.mit.edu/resources/KEMAR.html
